# Past seawater experience enhances seawater adaptability in medaka, *Oryzias latipes*

**DOI:** 10.1186/s40851-016-0047-2

**Published:** 2016-06-15

**Authors:** Hiroshi Miyanishi, Mayu Inokuchi, Shigenori Nobata, Toyoji Kaneko

**Affiliations:** Department of Aquatic Bioscience, Graduate School of Agricultural and Life Sciences, The University of Tokyo, Bunkyo, Tokyo 113-8657 Japan; Department of Biology, Keio University, 4-1-1, Hiyoshi, Kohoku, Yokohama, Kanagawa 223-8521 Japan; Atmosphere and Ocean Research Institute, The University of Tokyo, Kashiwa, Chiba 277-8564 Japan

**Keywords:** Seawater adaptation, Osmoregulation, Ionocytes, Ion secretion, Epigenetic regulation, Medaka

## Abstract

**Background:**

During the course of evolution, fishes have acquired adaptability to various salinity environments, and acquirement of seawater (SW) adaptability has played important roles in fish evolution and diversity. However, little is known about how saline environments influence the acquirement of SW adaptability. The Japanese medaka *Oryzias latipes* is a euryhaline species that usually inhabits freshwater (FW), but is also adaptable to full-strength SW when transferred through diluted SW. In the present study, we examined how past SW experience affects hyposmoregulatory ability in Japanese medaka.

**Results:**

For the preparation of SW-experienced fish, FW medaka were acclimated to SW after pre-acclimation to 1/2 SW, and the SW-acclimated fish were transferred back to FW. The SW-experienced fish and control FW fish (SW-inexperienced fish) were transferred directly to SW. Whereas control FW fish did not survive direct transfer to SW, 1/4 of SW-experienced fish adapted successfully to SW. Although there were no significant differences in blood osmolality and plasma Na^+^ and Cl^−^ concentrations between SW-experienced and control FW medaka in FW, increments in these parameters following SW transfer were lower in SW-experienced fish than in control FW fish. The gene expression of SW-type Na^+^, K^+^-ATPase (NKA) in the gills of SW-experienced medaka increased more quickly after direct SW transfer compared with the expression in control FW fish. Prior to SW transfer, the density of NKA-immunoreactive ionocytes in the gills was higher in SW-experienced fish than in control FW fish. Ionocytes expressing CFTR Cl^−^ channel at the apical membrane and those forming multicellular complexes, both of which were characteristic of SW-type ionocytes, were also increased in SW-experienced fish.

**Conclusion:**

These results indicate that past SW experience enhances the capacity of Na^+^ and Cl^−^ secretion in ionocytes and thus hypoosmoregulatory ability of Japanese medaka, suggesting the presence of epigenetic mechanisms involved in seawater adaptation.

**Electronic supplementary material:**

The online version of this article (doi:10.1186/s40851-016-0047-2) contains supplementary material, which is available to authorized users.

## Background

Fishes inhabit various salinity environments, such as freshwater (FW), brackish water (BW) and seawater (SW). In adapting to various salinity environments, fish have acquired osmotic adaptability during the course of evolution. Seawater environments account for more than 95 % in the aquatic environments [1]. Adaptation to SW plays important roles in evolution and species diversity in fish. However, little is known about how saline environments influence the acquirement of SW adaptability, which could be caused by genetic and/or epigenetic mechanisms.

The genus *Oryzias*, which includes both FW and SW species, is a good model for studying mechanisms of osmotic adaptation. In *Oryzias* species, SW adaptability correlates closely with distribution areas in their natural habitats. *O. marmoratus*, which inhabits highland FW lakes, completely lacks SW adaptability [2]. *O. javanicus*, which inhabits BW and SW [3], is fully adaptable to a wide range of salinity from FW to SW [2]. *O. latipes*, which is distributed in FW of South Asia [3] and sometimes found in BW [4], cannot survive direct transfer from FW to full-strength SW, although it is adaptable to SW after pre-acclimation to diluted SW (1/3–3/4 of SW) [2, H. Miyanishi, unpublished observations]. Therefore, *O. latipes* has intermediate SW adaptability among *Oryzias* species. In addition, inbred strains have been established in *O. latipes*, and those strains have theoretically no genetic difference among individuals [5]. Zebrafish is also a good fish model; however, it is a stenohaline fish which survives only in FW. Therefore, *O. latipes* is a powerful model for evaluating genetic and/or epigenetic mechanisms in osmoregulation.

Marine teleosts maintain their body fluid osmolality at approximately one-third the SW level by drinking SW and excreting Na^+^ and Cl^−^ from gills [6–10]. Since Na^+^ and Cl^−^ constitute more than 90 % of SW and blood osmolality, excretion of excess Na^+^ and Cl^−^ through ionocytes is of primary importance in adaptation of teleost fish to hyperosmotic SW environments. Among osmoregulatory organs and cells in teleosts, gill ionocytes (also referred to as mitochondrion-rich cells or chloride cells) are important not only in secreting Na^+^ and Cl^−^ in SW, but also in absorbing them in FW [7–9]. For euryhaline teleosts, the functional classification of ionocytes was proposed based on observations on the embryonic skin and yolk-sac membranes of Mozambique tilapia (*Oreochromis mossambicus*) and Japanese medaka (*O. latipes*) [11, 12]. Ionocytes in tilapia embryos were classified into four distinct types according to expressed ion-transporters and their localization: type I, showing only basolateral Na^+^/K^+^-ATPase (NKA); type II, basolateral NKA and apical Na^+^-Cl^−^ cotransporter 2 (NCC2); type III, basolateral NKA and Na^+^-K^+^-2Cl^−^ cotransporter 1a (NKCC1a) and apical Na^+^/H^+^ exchanger 3 (NHE3); and type IV, basolateral NKA and NKCC1a and apical cystic fibrosis transmembrane conductance regulator (CFTR) Cl^−^ channel [11]. In Japanese medaka, the classification of ionocytes is similar to that in tilapia, although there are some minor differences in ion transporters expressed in ionocytes between the two euryhaline species [12].

The purpose of this study is to examine whether or not past SW experience affects hyposmoregulatory ability in Japanese medaka. The survival rates were examined in SW-experienced and inexperienced (control) medaka after direct transfer from FW to SW. We found that SW adaptability was greatly enhanced by past SW experience in Japanese medaka. To further investigate the possible involvement of gill ionocytes in the enhanced SW adaptability, we also examined the effects of past SW experience on ionocytes by means of morpho-functional analyses.

## Methods

### Fish

Mature Japanese medaka of d-rR strain, which were provided from National Bio-Resource Project (National Institute for Basic Biology, Okazaki, Japan), were maintained in FW at 26 °C in a re-circulating system under a 14:10-h light–dark photoperiod. All animal experiments were conducted according to the Guideline for Care and Use of Animals approved by the committees of the University of Tokyo.

### Preparation of SW-acclimated and SW-experienced medaka and their survival after SW transfer

Medaka that were kept in FW and had never experienced SW after birth were used as control fish. For preparation of SW-acclimated medaka, fish were transferred from FW to SW after pre-acclimation to 1/2 SW for 1 day and kept in SW for 4 weeks. For preparation of SW-experienced medaka, those SW-acclimated fish were transferred back to FW and kept in FW for 4 weeks prior to the following experiments. To prevent handling stress, all acclimation procedures were performed by changing the water. Then, the water in the tank of FW medaka groups also was renewed. The SW-experienced medaka and control FW medaka (eight individuals each) were transferred directly to SW. The SW-transfer experiment was repeated three times. The survival was monitored for more than 24 h after SW transfer. The death of fish was defined as the stopped movement of the operculum.

### Measurement of blood osmolality and plasma Na^+^ and Cl^−^ concentrations

The SW-experienced medaka and control FW medaka were transferred directly to SW, and the surviving fish were sampled at 0 h (FW), 3 and 6 h after SW transfer. After anesthesia in 0.05 % (*v/v*) 2-phenoxyethanol (Wako, Osaka, Japan), the blood (less than 6 μl) was collected from the heart with a glass capillary. The collected blood was immediately transferred into a heparinized hematocrit tube (Terumo, Tokyo, Japan), which was sealed to prevent evaporation. The blood osmolality was measured as previously described [13, 14]. In brief, approximately 4 μl of the blood was applied onto a sample disc (6 mm in diameter) made of #1 filter paper (Whatman, Kent, UK), and the osmolality was measured with a vapor pressure osmometer (model 5520, Wescor, Logan, UT) on the same day of blood sampling. For the measurement of plasma Na^+^ and Cl^−^ concentrations, the collected blood in a heparinized hematocrit tube was centrifuged at 10,000 × g for 10 min at 4 °C. One microlitre plasma was collected from the centrifuged blood sample with 1-μl Microcaps (Drummond scientific, Broomall, PA), and diluted with 1 ml of Milli-Q water. Plasma Na^+^ and Cl^−^ concentrations were determined with an ion analyzer (IA-200, TOA-DKK, Tokyo, Japan).

### Real-time quantitative PCR (real-time qPCR)

Total RNA was extracted from the gills of SW-experienced medaka and control FW medaka at 0 h (FW), 3, 6 and 24 h (only SW-experienced medaka) after SW transfer with ISOGEN (Nippongene, Toyama, Japan). To eliminate genomic contamination, all total RNA samples were treated with 1 unit of TURBO DNase (Applied Biosystems; Life Technologies, Gaithersburg, MD) according to the standard protocol. One microgram of DNase-treated total RNA was reverse-transcribed using High-Capacity cDNA Reverse Transcription Kit (Applied Biosystems). Specific regions of medaka NKAα-subunit 3b, NKCC1a and CFTR were identified in our previous report [14]. Six NKAα-subunit isoforms were identified in a previous report (Bollinger et al., 2016) [15]. Partial cDNAs were cloned using specific primer sets, sequenced and used as standards (Additional file 1: Table S1). The amount of plasmid was determined in triplicate with a Nano-drop 1000 (Thermo Fisher Scientific, Waltham, MA). The plasmid was serially diluted, and a standard curve was generated. The amount of mRNA was determined by real-time qPCR assay using a Light-cycler 480 real-time PCR system (Roche Diagnostics, Mannhein, Germany). As an internal control, expression levels were subsequently normalized to that of elongation factor 1α (EF1α; AB013606) and expressed as the quantity relative to EF1α. Primer sets for real-time qPCR were designed using Primer Express software (Applied Biosystems) (Additional file 1: Table S1). All PCR reactions were conducted in a 20-μl mixture consisting of 10 μl Light-cycler 480 SYBR green I master (Roche Diagnostics), 0.12 μl each of 50 μM forward and reverse primers and 5 μl plasmid standard or diluted cDNA template. The specificity of PCR for each target gene was confirmed by dissociation curve analysis with a Light-cycler 480 real-time PCR system (Roche Diagnostics). Each primer set for NKA α-subunit isoforms did not amplify the other isoforms using standard templates, indicating the specificity of PCR for NKA isoforms.

### Whole-mount immunocytochemistry and morphometrical analyses of gill ionocytes

For the detection of NKA-immunoreactive ionocytes, we used a rabbit polyclonal antiserum raised against a synthetic peptide corresponding to part of the highly conserved region of the NKA α-subunit [16]. The antibody for CFTR was a mouse monoclonal antibody against 104 amino acids at the carboxyl terminus of human CFTR (R&D Systems, Boston, MA).

The gills of SW-acclimated medaka (4-week acclimation) before re-acclimation to FW and control FW medaka were fixed in 4 % paraformaldehyde (PFA) in 0.1 M phosphate buffer overnight at 4 °C, and then stored in 70 % ethanol at 4 °C. The gills of SW-experienced and control FW medaka were sampled at 0 h (FW), 3, and 6 h after SW transfer, fixed in 4 % PFA overnight at 4 °C, and then stored in 70 % ethanol at 4 °C. The gill samples were rehydrated in 10 mM phosphate-buffered saline (PBS) containing 0.2 % Triton X-100 (PBST) for 1 h, incubated with PBST containing 10 % normal goat serum, 0.1 % bovine serum albumin, 0.02 % keyhole limpet hemocyanin, 0.05 % Triton X-100 and 0.01 % sodium azide (NBPBST) for 2 h at room temperature, and then incubated with a mixture of anti-NKA and anti-CFTR for 2 days at 4 °C. Final dilutions of anti-NKA and anti-CFTR were 1:500 and 1:250, respectively. The samples were then incubated overnight at 4 °C with a mixture of goat anti-rabbit IgG labeled with Alexa Fluor 488 and goat anti-mouse IgG labeled with Alexa Fluor 555 (Invitrogen Life Technologies), both diluted 1:500 with NBPBST. The samples were washed in PBST, and incubated with DAPI (5 μg/ml) in PBS for 1 h at room temperature. After washing in PBST, the samples were observed and photographed with a confocal laser scanning microscope (C1, Nikon, Tokyo, Japan). Z-stacks were rendered in three dimensions with EZ-C1 software (Nikon). The wavelengths of excitation and recorded emission for each fluorescent dye were as follows: Alexa Fluor 555, 543 and 605/75 nm; Alexa Fluor 488, 488 and 515/30 nm; and DAPI, 405 and 450/35 nm. The cell numbers were counted for NKA-immunoreactive cells (all ionocytes) and those immunoreactive to both CFTR and NKA (SW-type ionocytes). According to their morphological characteristics, ionocytes were largely classified into single cells and multicellular complexes, which were readily distinguishable on the basis of Z-stack images. The numbers were also counted for single and multicellular complex ionocytes. The size of gill ionocytes in FW and SW-acclimated medaka was measured using ImageJ (http://rsb.info.nih.gov/ij/).

### Statistical analyses

Data are expressed as means ± SEM. Data were analyzed by one-way ANOVA, and Tukey-Kramer multiple comparison test was used for time-course changes in real-time qPCR, blood osmolality, plasma Na^+^ and Cl^−^ concentrations. Student’s *t* test was used for the survival rate, blood osmolality, gene expression and cell numbers. Significance was set at *P* < 0.05. All statistical analyses were performed using Kyplot 5.0 (Kyenslab, Inc., Tokyo, Japan).

## Results

### Survival rates of SW-experienced and control FW medaka after direct SW transfer

**Fig. 1:**
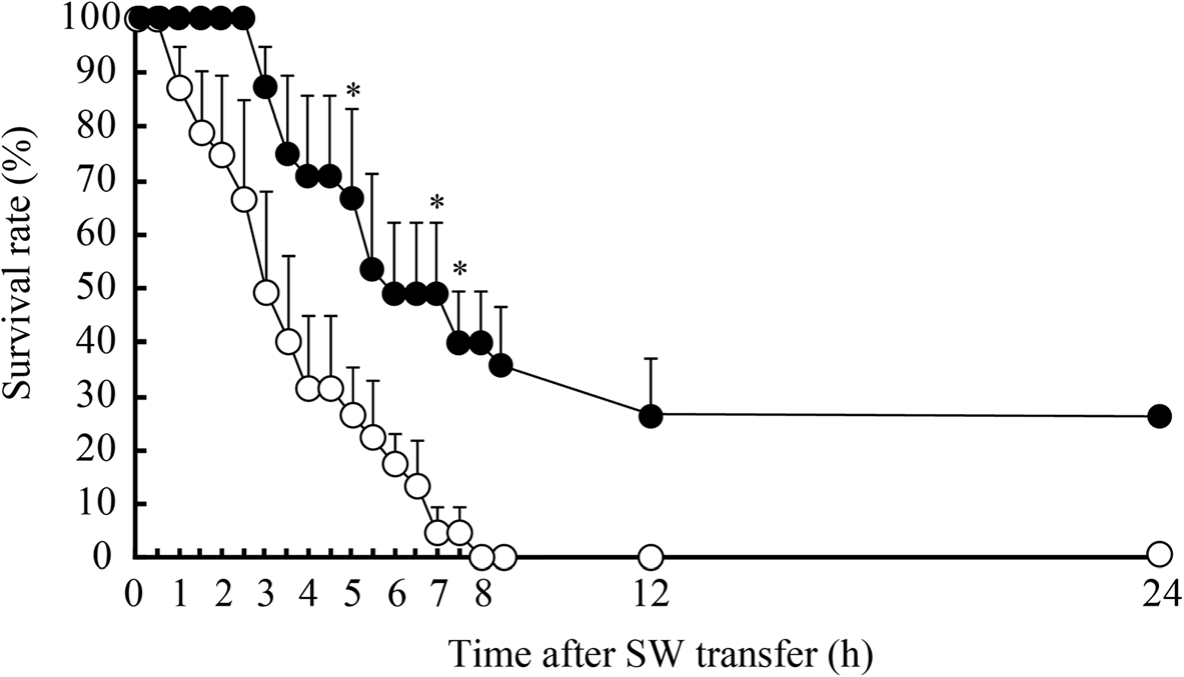
Changes in survival rates after direct sweater (SW) transfer of SW-experienced medaka (*solid circles*) and control freshwater (FW) medaka (*open circles*). Experiments were repeated three times (*n* = 3). Values are means ± SEM. *Asterisks* indicate significant differences between the two groups at the same time points between 0 and 7.5 h (two-sided Student’s *t* test, *P < 0.05)

Approximately 26 % of SW-experienced medaka survived direct transfer to SW for more than 24 h, whereas the rest of the fish died between 3 and 12 h after transfer (Fig. 1). In contrast, all control FW medaka, which had not experienced SW before, died by 8 h after SW transfer (Fig. 1). The average survival duration in SW was 3.7 ± 0.4 h for control FW medaka and 11.6 ± 2.0 h for SW-experienced medaka (during a monitoring period of 24 h), which were significantly different from each other (*P* < 0.001).

### Blood osmolality and plasma Na^+^ and Cl^−^ concentrations

**Fig. 2:**
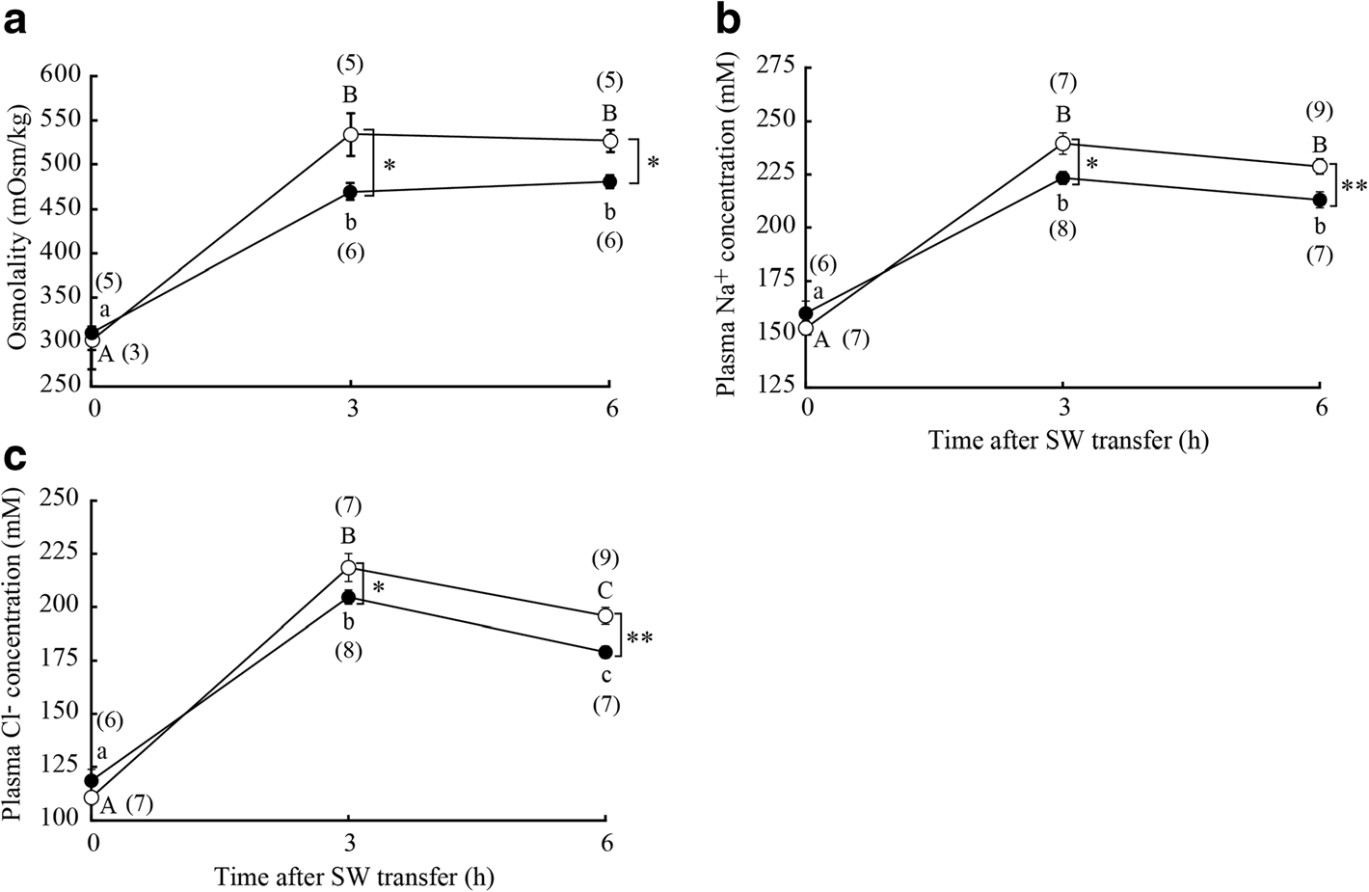
Changes in blood osmolality (**a**) and plasma Na^+^ (**b**) and Cl^−^ (**c**) concentrations after direct SW transfer of SW-experienced medaka (*solid circles*) and control FW medaka (*open circles*). Values are means ± SEM. *Numerals in parentheses* indicate the number of samples examined. *Different letters* indicate significant differences within the same group (ANOVA, Tukey-Kramer multiple comparison test, *P* < 0.05). *Asterisks* indicate significant differences between the two groups at the same time points (two-sided Student’s *t* test, *P < 0.05 and **P < 0.01)

In FW (before SW transfer), there was no significant difference in blood osmolality between SW-experienced and control FW medaka (Fig. 2a). Blood osmolality increased after direct SW transfer in both groups; however, the increment in blood osmolality was significantly lower in SW-experienced medaka than in control FW medaka (Fig. 2a). Blood osmolality of control FW medaka increased to more than 500 mOsm/kg at 3 and 6 h after SW transfer, whereas it stayed under 500 mOsm/kg in SW-experienced medaka. Plasma Na^+^ and Cl^−^ concentrations, which showed no significant differences between SW-experienced and control FW medaka in FW, increased significantly after SW transfer (Fig. 2b and c). Increased plasma Na^+^ and Cl^−^ concentrations after SW transfer were significantly lower in SW-experienced medaka than in control FW medaka (Fig. 2b and c). Plasma Cl^−^ concentration of SW-experienced and control FW medaka was increased at 3 h, followed by slight but significant decrease at 6 h (Fig. 2c). Such a decrease at 6 h was not seen in plasma Na^+^ levels in either group.

### Gene expressions of ion transporters in the gills

**Fig. 3:**
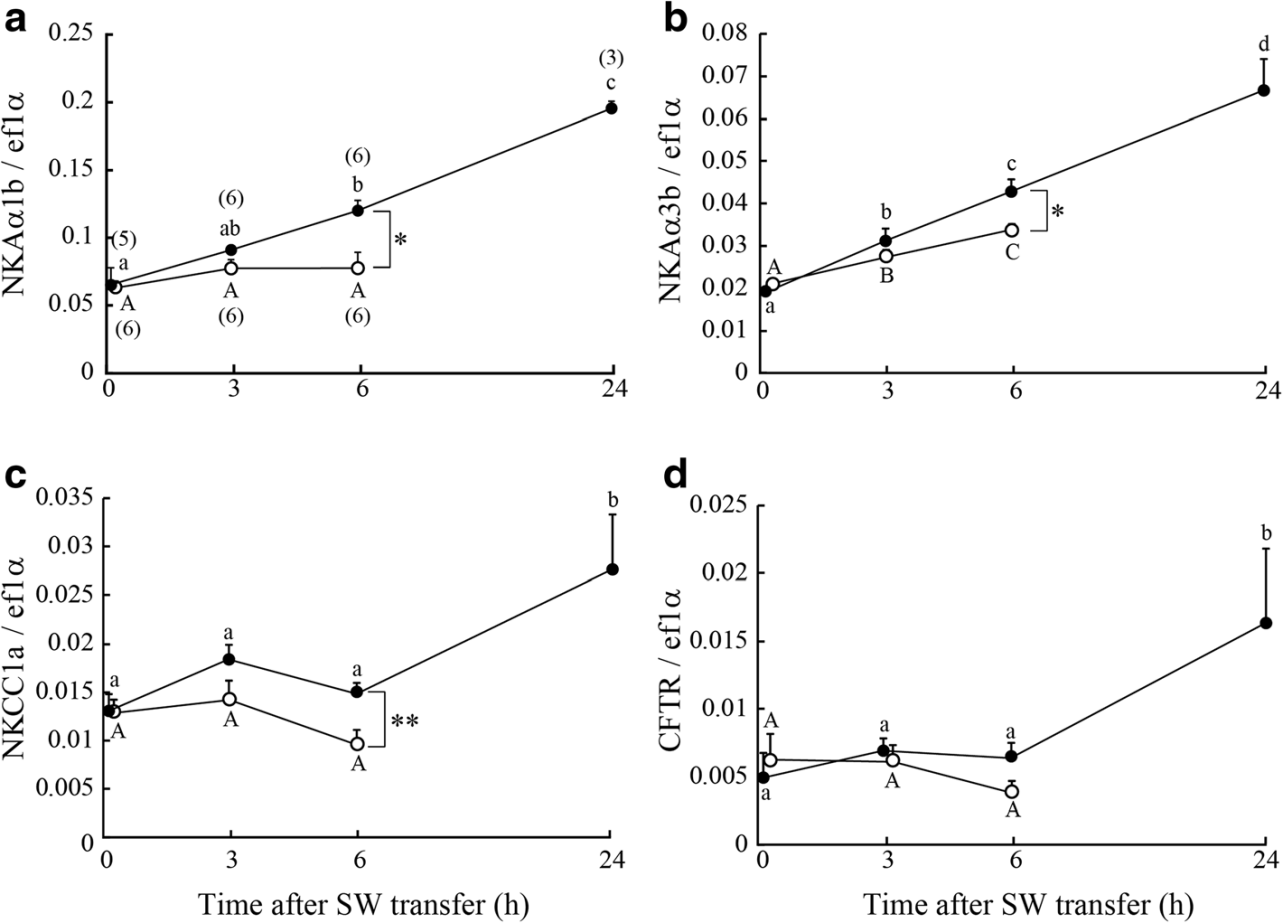
Changes in gene expressions of Na^+^/K^+^-ATPase α-subunit 1b (NKAα1b, **a**), NKAα3b (**b**) Na^+^-K^+^-2Cl^−^ cotransporter 1a (NKCC1a, **c**), and cystic fibrosis transmembrane conductance regulator Cl^−^ channel (CFTR, **d**) after direct SW transfer of SW-experienced medaka (*solid circles*) and control FW medaka (*open circles*). Values are means ± SEM. *Numerals in parentheses* indicate the number of samples examined. *Different letters* indicate significant differences within the same group (ANOVA, Tukey-Kramer multiple comparison test, *P* < 0.05). *Asterisks* indicate significant differences between the two groups at the same time points (two-sided Student’s *t* test, *P < 0.05 and **P < 0.01)

In FW before SW transfer, there were no significant differences in gene expressions of NKA, NKCC1a and CFTR in the gills between SW-experienced and control FW medaka (Fig. 3). Among six NKA α-subunit isoforms identified in *O. latipes*, NKAα1b and NKAα3b mRNA were expressed at the highest level (Fig. 3a, b and Additional file 2: Figure S1). After direct SW transfer, the gene expression of NKAα1b and NKAα3b increased at a higher rate in SW-experienced medaka than in control FW ones (Fig. 3a and b). In SW-experienced medaka, the gene expression of NKAα1b was increased at 6 h after SW transfer in SW-experienced medaka (Fig. 3a). The expression of NKAα3b was increased at 3 and 6 h after SW transfer, and became approximately 3-fold higher at 24 h than at 0 h (FW) in SW-experienced medaka (Fig. 3b). Although the expression of NKCC1a was not significantly increased at 3 and 6 h in both groups, it was significantly higher in SW-experienced medaka than in control FW ones at 6 h (Fig. 3c). Thereafter, the NKCC1a expression was significantly increased at 24 h in SW-experienced medaka (Fig. 3c). There was no significant difference in the expression of CFTR at 3 and 6 h between the two groups; however, it was significantly increased in SW-experienced at 24 h (Fig. 3d).

### Morphometrical analyses of gill ionocytes

**Fig. 4:**
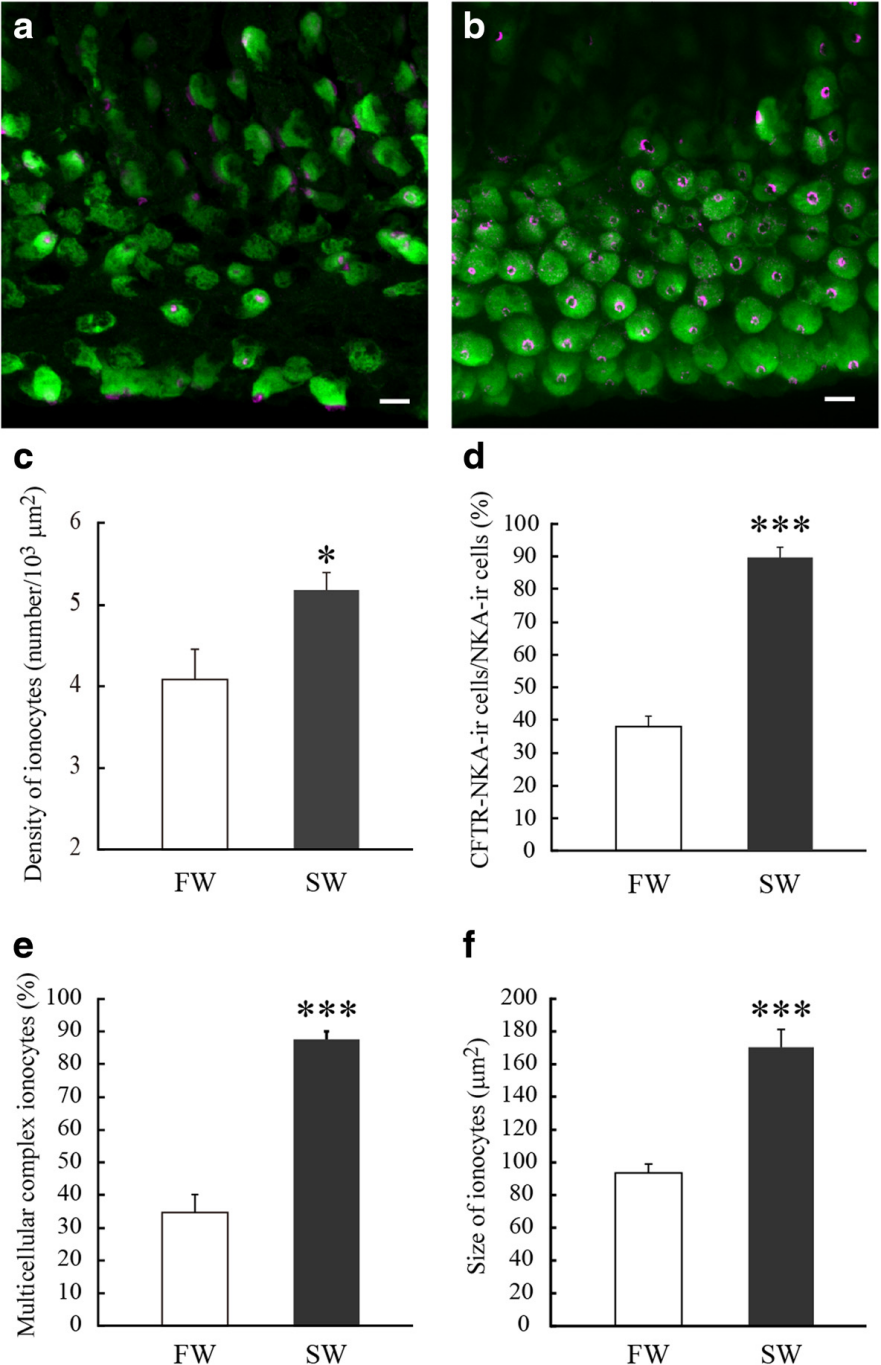
**a**, **b** Double immunofluorescence staining for NKA (*green*) and CFTR (*magenta*) in the gills of FW (**a**)- and SW (**b**)-acclimated medaka. **c**–**e** The density of ionocytes (**c**), ratio of CFTR-immunoreactive ionocytes to NKA-immunoreactive ionocytes (**d**), ratio of multicellular complex ionocytes to total ionocytes (**e**) and size of ionocytes (**f**) in the gills of FW- and SW-acclimated medaka. Values are means ± SEM (*n* = 6). *Asterisks* indicate significant differences between the two groups (two-sided Student’s *t* test, *P < 0.05 and ***P < 0.001)

**Fig. 5:**
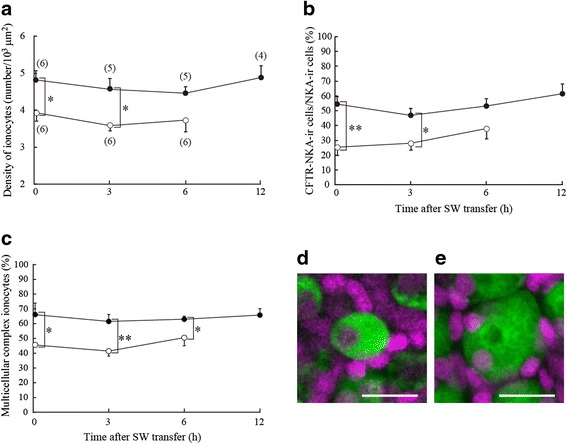
**a**–**c** Changes in the density of ionocytes (**a**), ratio of CFTR-immunoreactive ionocytes to NKA-immunoreactive ionocytes (**b**), and ratio of multicellular complex ionocytes to total ionocytes (**c**) after direct SW transfer of SW-experienced medaka (*solid circles*) and control FW medaka (*open circles*). Values are means ± SEM. *Numerals in parentheses* indicate the number of samples examined. There are no significant differences in those parameters within the same group (ANOVA, Tukey-Kramer multiple comparison test, *P* < 0.05). *Asterisks* indicate significant differences between the two groups at the same time points (two-sided Student’s *t* test, *P < 0.05 and **P < 0.01). **d**, **e** Micrographs of representative single (**d**) and multicellular complex (**e**) ionocytes in the gills. Whole mount samples of gill filaments were immunostained with anti-NKA (*green*) and counterstained with DAPI (*magenta*). *Scale bar*, 10 μm

First, ionocytes in the gills of FW- and SW-acclimated medaka were subjected to morphometrical analyses. The density of NKA-immunoreactive ionocytes in the gills was significantly higher in SW medaka (5.18 ± 0.22 cells/10^3^ μm^2^) than in FW ones (4.09 ± 0.38 cells/10^3^ μm^2^) (Fig. 4a, b and c). Approximately 90 % of ionocytes in SW gills expressed CFTR on the apical membrane, which is characteristic of SW-type (NaCl-secreting type) ionocytes (Fig. 4b and d). Meanwhile, CFTR was also expressed on the apical membrane of approximately 39 % of ionocytes in the gills of FW fish (Fig. 4a and d). Approximately 90 % of ionocytes in SW gills formed multicellular complexes, whereas the ratio was significantly lower in FW gills (approximately 35 %) (Fig. 4e). The size of gill ionocytes in SW was larger than that in FW (Fig. 4a, b and f). Typical single and multicellular complex ionocytes are shown in Fig. 5d and e, respectively.

The density of ionocytes in the gills was significantly higher in SW-experienced medaka (4.81 ± 0.26 cells/10^3^ μm^2^) than in control FW medaka (3.93 ± 0.23 cells/10^3^ μm^2^) at 0 h (before SW transfer) (Fig. 5a). Approximately 55 % of ionocytes in SW-experienced medaka gills expressed CFTR on the apical membrane in FW, being significantly higher than in control FW medaka (approximately 25 %) (Fig. 5b). Ionocytes forming multicellular complexes accounted for approximately 65 % of gill ionocytes in SW-experienced medaka, which was significantly greater than in control FW medaka (approximately 45 %) (Fig. 5c). In both groups, there were no significant changes in the density of ionocytes and the ratios of CFTR-immunoreactive and multicellular complex ionocytes after direct SW transfer (Fig. 5a, b and c).

## Discussion

In the present study, we found that some SW-experienced medaka survived direct SW transfer, whereas all SW-inexperienced medaka (control FW medaka) died after SW transfer. This result indicates that past experience of hyperosmotic environment enhances SW adaptability, suggesting the presence of epigenetic mechanisms involved in SW adaptation. In contrast to the euryhaline medaka *O. latipes*, FW stenohaline fishes such as *O. marmoratus*, goldfish and zebrafish lack SW adaptability [8, 17]. In those FW stenohaline species, it seems that the osmoregulatory ability has been genetically fixed during their evolution. In recent years, epigenetic regulatory mechanisms have been studied intensively in mammals, indicating that DNA methylation and histone modification are involved in such epigenetic changes. In vertebrates including mammals, however, little is known about epigenetic regulatory mechanisms related to osmoregulation, which should be subject to future investigation.

Ionocytes in the gills are important in maintaining the body fluid homeostasis, absorbing and excreting Na^+^ and Cl^−^ in FW and SW, respectively [7–9]. Therefore, the functional plasticity of ionocytes could be a key factor to survival following transfer from FW to SW or vice versa. In the present study, there were no significant differences in blood osmolality and plasma Na^+^ and Cl^−^ levels between SW-experienced and control FW medaka before SW transfer. The gene expressions of NKA, NKCC1a and CFTR also showed no differences between the two groups. These results indicated that, in SW-experienced medaka, the 4-week acclimation to FW was long enough to restore those physiological parameters to FW levels. About one quarter of SW-experienced medaka survived direct transfer from FW to SW. In those surviving fish, blood osmolality did not exceed 500 mOsm/kg. This is in sharp contrast with control FW medaka, in which blood osmolality was increased to more than 500 mOsm/kg after SW transfer. Considering that all control FW medaka failed to survive SW transfer, these results indicated that a blood osmolality around 500 mOsm/kg could be a critical limit for survival in SW.

The present study revealed that changes in blood osmolality and plasma Na^+^ and Cl^−^ levels showed similar profiles, being increased after direct SW transfer. Among those parameters, however, the plasma Cl^−^ concentration in both SW-experienced and control FW medaka showed some recovery at 6 h from the greatly elevated levels measured at 3 h. This result indicated that Cl^−^ was more actively secreted than Na^+^ via ionocytes at 6 h. Shen et al. [18] observed Na^+^ and Cl^−^ secretion from individual ionocytes in medaka larvae, showing that Na^+^ secretion via ionocytes occurred by 4 h after transfer from FW to SW and was fully developed in 5 h. Meanwhile, Cl^−^ secretion occurred by 30 min after transfer from FW to SW, and was fully developed in 2 h. Therefore, Cl^−^ secretion occurs much earlier than Na^+^ secretion after SW transfer, which may result in more rapid restoration of plasma Cl^−^ than Na^+^.

The present study showed that CFTR was expressed at the apical side of gill ionocytes in FW medaka, although CFTR is assumed to act as a Cl^−^-exit site in SW-type ionocytes. In general, FW fish reduce Cl^−^ secretion from ionocytes and actively absorb Cl^−^ through NCC-expressing ionocytes in the gills [7, 8, 19]. In FW-acclimated Mozambique tilapia, CFTR expression was not detected by immunocytochemistry [11, 20]. A previous report showed that Cl^−^ secretion via ionocytes in SW-acclimated medaka larvae was reduced 30 min after FW transfer, and decreased to the same levels as FW-acclimated larvae by 3 h [18]. CFTR is a member of the ATP-binding cassette (ABC) transporter family of membrane proteins [21, 22]. Protein kinase A (PKA) phosphorylation of the R region in the CFTR structure is important in opening the CFTR channel [23]. It is highly possible that dephosphorylation of CFTR is involved in immediate inhibition of Cl^−^ secretion from ionocytes [24]. Another possibility is that apical pits of ionocytes could be physically closed by adjacent pavement cells to prevent Cl^−^ secretion, while CFTR remains on the apical membrane of ionocytes. It has been reported that apical pits of ionocytes are closed by hypotonic shock (FW transfer) in SW-acclimated killifish, *Fundulus heteroclitus* [25].

We found that the density of ionocytes was higher in SW-experienced medaka than in control FW ones, despite the fact that the fish in both groups were reared in FW. This finding suggests that SW experience induced the increment in the ionocyte number, and, more importantly, that the increased number of ionocytes was maintained even after FW re-acclimation. In this study, SW experience also increased CFTR-expressing (SW-type) ionocytes and multicellular complex ionocytes. The formation of multicellular complexes is necessary to form a leaky junction between ionocytes and accessory cells [7–9]. In SW fish, Na^+^ is secreted to the intercellular space via highly expressed NKA on the basolateral membrane, and concentrated more highly than that in SW. The concentrated Na^+^ is secreted through the paracellular pathway and the leaky junction formed between ionocytes and accessory cells [9]. The previous report showed that Na^+^ was secreted only from multicellular complexes of ionocytes, whereas Cl^−^ was secreted from both single and multicellular complex ionocytes [18]. This finding clearly demonstrated that the formation of multicellular complexes is necessary for Na^+^ secretion. As described above, the development of Cl^−^ secretion is followed by that of Na^+^ secretion in medaka transferred from FW to SW [18]. Since CFTR was already expressed on the apical membrane of some ionocytes even in FW, Cl^−^ secretion could begin shortly after SW transfer.

From this study, we conclude that blood osmolality likely exceeded the permissible physiological range for survival following SW transfer of control FW medaka, whereas blood osmolality in SW-experienced medaka stayed within the range. Most probably, the increased number of multicellular complexes of ionocytes is particularly important to permit efficient Na^+^ secretion in SW-experienced medaka. In contrast, the formation of multicellular complexes and leaky junctions in the gills of SW-experienced medaka are not favorable to the maintenance of body fluid homeostasis in FW. The junctions formed between ionocytes and adjacent accessory cells are shallow and leaky in SW-acclimated killifish and Mozambique tilapia, whereas the junctions between ionocytes and pavement cells are deep and tight in FW-acclimated fish [26, 27]. In SW-experienced medaka, the increased number of multicellular complexes and CFTR-expressing ionocytes would greatly expand capability for SW adaptation, despite any apparent disadvantages for FW adaptation. In the present study, no difference was observed in mRNA expression of NKAα1b and α3b in FW gills in SW-experienced and FW control medaka. Expression and activity of NKA are often correlated directly with salinity [28, 29]. The total NKA activity in the gills was likely to be regulated within a required level despite the increased density of ionocytes in SW-experienced medaka. Following transfer to SW, however, the expressions of NKAα1b and α3b in the gills increased more rapidly in SW-experienced medaka than in control FW ones. The rapid increase in NKAα1b and α3b expression in SW-experienced medaka could also be attributed to the increased density of ionocytes.

Even for the SW-experienced medaka experimental group, not all fish survived direct transfer to SW. In contrast, FW medaka are adaptable to SW after stepwise transfer through dilute SW. This implies that stepwise increase in environmental salinity allows fish the time needed to develop SW adaptability. Those medaka transferred directly to SW have insufficient time to prepare for adaptation to the hyperosmotic environment despite the increased potential for SW adaptability. The enhanced potential for SW adaptability of SW-experienced medaka may especially facilitate survival during the early phase following SW transfer, and this may further improve subsequent successful adaptation to SW. In this study, we showed for the first time that past SW experience enhances the capacity of SW adaptation, suggesting the presence of epigenetic mechanisms involved in the differentiation of ionocytes.

## Conclusions

We found that a portion of SW-experience medaka survived direct transfer from FW to SW, whereas all FW fish (SW-inexperienced fish) died after direct transfer to SW. Past SW experience increased the numbers of ionocytes, those expressing CFTR Cl^−^ channel, and those forming multicellular complexes in the gills. Therefore, the past SW experience enhanced the ability of Na^+^ and Cl^−^ secretion in ionocytes, resulting in increased hyposmoregulatory ability of Japanese medaka. These results suggest the presence of epigenetic mechanisms involved in seawater adaptation.

## Abbreviations

SW, seawater; FW, freshwater; BW, brackish water; NKA, Na^+^, K^+^-ATPase; NCC2, Na^+^-Cl^−^ cotransporter 2; NKCC1a, Na^+^-K^+^-2Cl^−^ cotransporter 1a; NHE3, Na^+^/H^+^ exchanger 3; CFTR, cystic fibrosis transmembrane conductance regulator; EF1α, elongation factor 1α

## Additional files

Additional file 1: Table S1. Primers used for cloning and real-time qPCR analyses. (XLSX 11 kb) Additional file 2: Figure S1. Changes in gene expressions of Na^+^/K^+^-ATPase α-subunits 1a (A), α1c (B), α2 (C) and α3a (D) after direct SW transfer of SW-experienced medaka (solid circles) and control FW medaka (open circles). Values are means ± SEM. Numerals in parentheses indicate the number of samples examined. Different letters indicate significant differences within the same group (ANOVA, Tukey-Kramer multiple comparison test, *p* < 0.05). Asterisks indicate significant differences between the two groups at the same time points (two-sided Student’s *t* test, **p* < 0.05 and ***p* < 0.01). (TIF 5121 kb)
