# Supplementary figures and images for: Past seawater experience enhances seawater adaptability in medaka, Oryzias latipes
Source: Zoological Lett. 2016 Jun 15;2:12. doi: 10.1186/s40851-016-0047-2 (PMC4908718; doi:10.1186/s40851-016-0047-2)

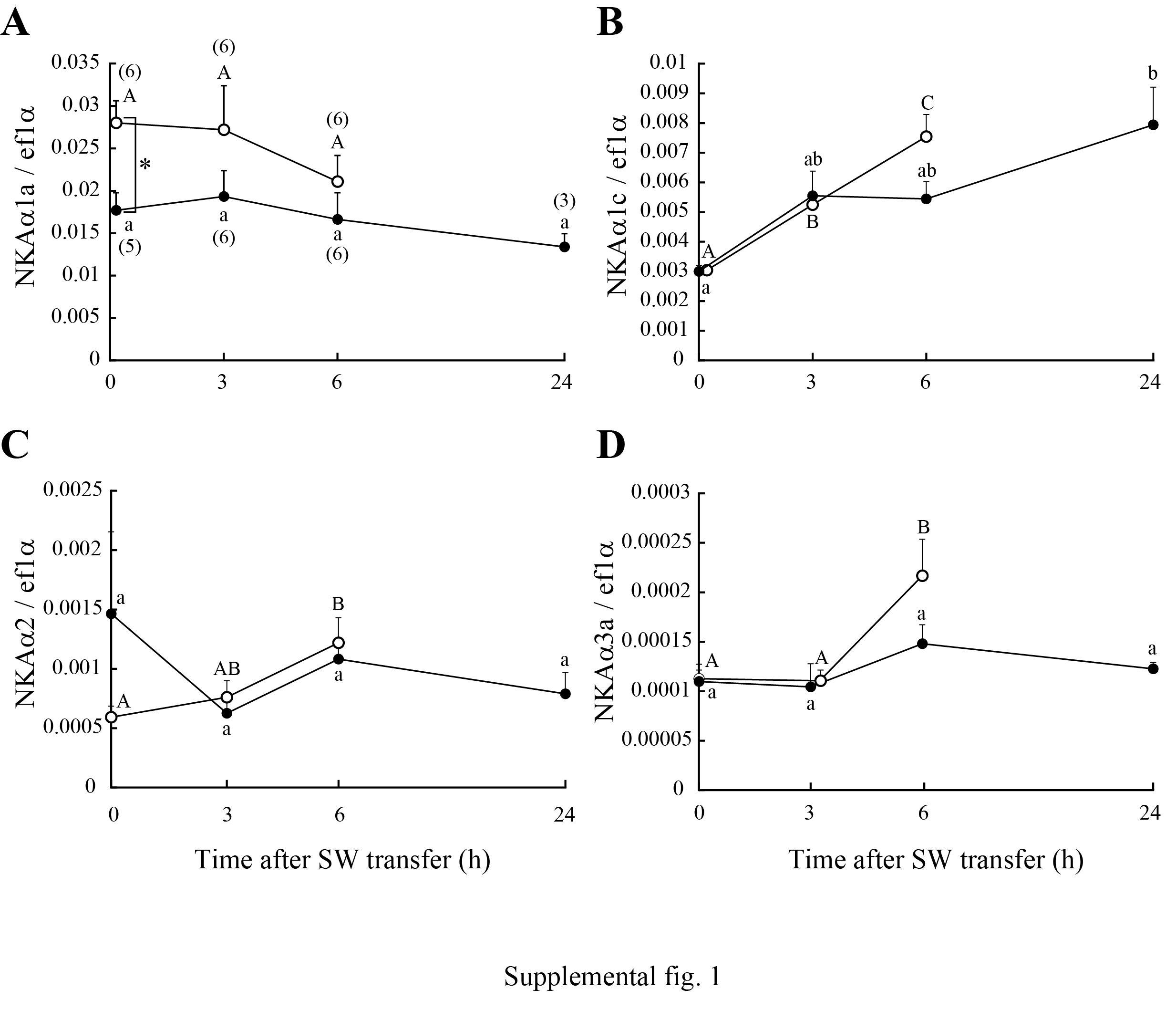

Supplement: Additional file 2: Figure S1. — Changes in gene expressions of Na+/K+-ATPase α-subunits 1a (A), α1c (B), α2 (C) and α3a (D) after direct SW transfer of SW-experienced medaka (solid circles) and control FW medaka (open circles). Values are means ± SEM. Numerals in parentheses indicate the number of samples examined. Different letters indicate significant differences within the same group (ANOVA, Tukey-Kramer multiple comparison test, p < 0.05). Asterisks indicate significant differences between the two groups at the same time points (two-sided Student’s t test, *p < 0.05 and **p < 0.01). (TIF 5121 kb) [file 40851_2016_47_MOESM2_ESM.tif]
